# Awareness of health risks associated with smokeless tobacco use among users in Pretoria

**DOI:** 10.4102/safp.v64i1.5560

**Published:** 2022-11-11

**Authors:** Tombo Bongongo, Yusuf Jeewa, Doudou K. Nzaumvila, Indiran Govender

**Affiliations:** 1 Department of Family Medicine & Primary Health Care, School of Health, Sefako Makgatho Health Sciences University, Pretoria, South Africa; 2 Ramotse Clinic, Tshwane Health District, Tshwane, South Africa

**Keywords:** awareness, health risks, smokeless tobacco use, smokeless tobacco users, Pretoria, South Africa

## Abstract

**Background:**

Smokeless tobacco (ST) refers to all tobacco-containing products that are not smoked but rather consumed through other means. Contrary to the popular belief that ST products are safe, the use of such products exposes users to health risks. To assess the awareness of health risks associated with ST use among users in a Pretoria community, the study was conducted in Ramotse community, located in Tshwane region 2, Gauteng, South Africa.

**Methods:**

This was a cross-sectional design, using a piloted, structured and self-administered questionnaire.

**Results:**

Of 479 participants with a mean age of 43 years (ranging from 18 to 89 years), most were in the age group 30–39 years (148; 31.6%), followed by ≥ 50 years (138; 29.4%). There were more females (371; 77.5%), more unemployed (263; 54.9%), married (236; 49.7%), had reached the secondary level of education (270; 56.4%), did not have any chronic illness (274; 57.2%), used snuff by nose (338; 70.6%), and were unaware of health risks associated with ST use (452; 94.4%).

**Conclusion:**

This study has demonstrated a poor awareness of health risks associated with ST use among the users in a Pretoria community. As a result, health education at various levels of the community (clinic, schools, ward-based outreach team or WBOT, etc.) could be one strategy for resolving the problem.

## Introduction

Smokeless tobacco (ST) refers to all tobacco-containing products which are not smoked. Existence of an association seems to have been established between the use of ST products and some health risks. Although exposure to these products produces a lower level of harmful chemicals among users than is the case with cigarette smoking, this does not make ST a safer substitute for cigarettes. Most of them contain cancer-causing chemicals and may expose users to addictive nicotine.^1^ Different types of ST products have been described, such as (1) chewing, oral or spitting tobacco, (2) snuff or dipping tobacco, (3) snus and (4) heated tobacco products. The first category is presented as loose leaves, plugs or twists of dried tobacco. It is placed between the gums (teeth) and the cheek and can be chewed. The second has two subtypes, dry and moist snuff. Dry snuff is a powder which is used by sniffing or inhaling the powder up the nose, while the moist snuff is placed between the lower lip or cheek and gum. The third type, snus, is packaged in small bags and placed between the gum and the mouth tissue.^1^ When it comes to heated tobacco products, often known as ‘heat-not-burn’, a typical electronic heating component is used to heat tobacco in specifically constructed sticks and capsules. The tobacco does not get hot enough to burn, but the heat releases nicotine that can be inhaled into the lungs.^1^ The World Health Organization (WHO) reports that more men are involved in tobacco use than women. There is a high rate of men using ST products globally.^2^ Contrary to the WHO perspective, a comparison between cigarette smoking and chewing tobacco (ST) in Bangladesh showed that chewing tobacco was common in females at around 22% of the sample compared to the 19% of male smokers. In addition, it was noted that chewing tobacco was more common in rural areas than in cities.^3^ In South Africa, this practice of ST, such as chewing or snuffing tobacco, was also noticed among the school learners. It affected more Grade 8 learners who were male than female, and there was a strong correlation between the grade in school, race, socioeconomic status, urbanisation and the prior use of tobacco (before age 10).^4^ Another South African study has shown that ST products are being used more frequently by South African women of reproductive age; they contain high levels of nicotine that could cause addiction and harm their health.^5^ Considering the geographic distribution of use of ST, most users are found in Southeast Asia; but the practice is also noted in Central Asia and in African countries such as Nigeria, Algeria, Sudan, and South Africa.^6^

To find out whether there is an increased risk of myocardial infarction and stroke among ST users in the United States and Sweden, a meta-analysis conducted in these two countries showed a higher risk of death from both health conditions; there is therefore an association between the use of ST products and the occurrence of fatal myocardial infarction and cerebrovascular accident (stroke).^7^ Smokeless tobacco products have addictive and carcinogenic properties, and contain high levels of nicotine and carcinogens. This is related to the use of Nicotiana rustica tobacco leaves; during storage nitrites are released in high amounts which react with tobacco alkaloids to produce nitrosamines (tobacco-specific nitrosamines [TSNA]), which are known to be carcinogenic. Other carcinogens, such as polycyclic aromatic hydrocarbons, aldehydes and phenols, will also be produced.^6^ In addition, the use of tobacco (smoking or smokeless) is associated with increased risk of other chronic and terminal diseases such as periodontal diseases, oral and pharyngeal cancers, erectile dysfunction, pregnancy-related issues such as stillbirth and low birthweight neonates, heart diseases, and more.^8^ Regarding the users’ demographics for this practice, it appeared that the majority were less than 30 years old, according to a Korean study.^9^ In a Bangladeshi study, current consumption of ST products among married women was associated with age (over 25 years), low education level, occupation and religion.^10^ Socially deprived groups are more exposed to the use of ST products, and the levels of knowledge about the harmful effects of this practice are low compared with such knowledge about cigarette smoking. The high demand for these products (ST) has been attached to its availability and cheap price.^6^ And in Sri Lanka, several workshops held among student nurses resulted in an enhanced knowledge and attitude towards the use of ST as well as to areca nut (a stimulant from areca palm).^8^

While assessing health knowledge and attitudes regarding ST consumption and its health effects among married women in a rural Bangladesh, it appeared that half of the participants believed that ST consumption had a positive impact on their health, and they attached to it some medicinal properties such as it being able to resolve stomach pains. However, on the other hand in the same area, some participants intended to quit the use of these products.^10^ Sadly, this is a false belief (from married women in a rural Bangladesh) that might encourage the usage of ST products. Similar to smoking, snus use in Sweden is associated with a higher risk of type 2 diabetes. When attempting harm reduction by switching from smoking snuff (snus) to using it as a smokeless product, this should be considered. Ten years of follow-up were conducted on two groups of male nondiabetics who used snus and cigarettes. The oral glucose tolerance test was used in the follow-ups to identify an elevated risk of developing diabetes mellitus in both groups. This led to the conclusion that, when used in large quantities, both snus and cigarettes put their consumers at an increased risk of developing diabetes mellitus. The effects of tobacco smoking on beta-cell activity may make it more common for people to develop diabetes mellitus.^11^ In Southeast Asian nations, ST products like ‘paan’ and ‘gutka’ have been popular. The usage of these products has been linked to conditions such as low birthweight, abortion, oral, throat, and esophageal malignancies, gum and tooth disease, dyspepsia, high blood pressure, dyslipidemia, diabetes mellitus and so on.^12^ While looking at the association between type 2 diabetes mellitus and ST use in Venezuela, the users of ‘chimo’, an ST product used in this country, were assessed during a cross-sectional study. There were more females in the sample, but it appeared that there was an elevated rate of ST use among males. The respondents using ST product had a lower body mass index, body fat and total cholesterol, and an elevated risk of type 2 diabetes mellitus. This study led to the conclusion that ‘chimo’ is associated with an elevated risk of type 2 diabetes mellitus and lower fat mass in Venezuela.^13^ More than 90% of the global population of adults who use ST products are concentrated in Southeast Asian and African nations with low- and low-middle incomes. They are more prevalent in the poorest rural areas of these regions; and males are more likely to use ST than females. Therefore, the burden of ST use prevails more in those regions of the globe.^14^ Similar findings were noted in another study where South and Southeast Asia seemed to have elevated rates of consumption of ST products. India was reported to have more than 50% of ST users in the region, with many lives lost because of oropharyngeal-oesophagus cancers and ischaemic heart disease associated with ST use. Almost 70% of these ST-related diseases are noted among men.^15^

In a cross-sectional study carried out in Lagos, Nigeria, among 400 participants, more than 50% were aware that ST use was harmful to health. However, more than half of the sample were unaware of some of the health risks, such as gum diseases, and lip and tongue cancers, associated with the use of ST products. Less than one-third of the participants had a good knowledge of the health risks associated with this habit. More than 50% felt that the habit was socially acceptable, while almost half of the sample mentioned that it was a waste of money.^16^ Given the popular rumour of ST products being a safer alternative to smoking cigarettes, a study was conducted in order to assess the heavy metals contained in the ST products frequently used in Nigeria. The assessment evaluated the concentration of each and every heavy metal hazard found in the ST products commonly used in the country, and different heavy metals, such as nickel, cobalt, chromium and cadmium, were found. The study did not find an apparent risk when analysing these metals individually, but a likely risk has to be considered while putting together the impact of all of these heavy metals; therefore, there is a potential risk for the users of these Nigerian heavy metals.^17^ Twenty-four percent of smokers were willing to switch to snuff on account of the likely role of reducing harm from smoking only if they are not used to skipping smoke-free laws related to reduced smoking, when trying to find the possibility of South African smokers switching to an imaginary reduced damage that can be provided by those products.^18^ While exploring the views on the practice of ST, interviews with traditional healers in the South African province of Limpopo were conducted to learn more about what they thought about ST usage and its potential health implications. The participants’ average age was 55 years, and they had an average of 17 years of healing experience. The participants considered the use of ST as essential in divination. Thirty-two percent of them, under the instruction of the ancestors, have prescribed this practice to their clients, while others who were even ST users themselves did not appreciate the danger of the recreational use of these products since it is addictive.^19^ In a qualitative study exploring attitudes towards the use of ST, the Northern province of South Africa, 22% of women and 11% of men revealed using snuff; and the most popular ST product was homemade snuff, which is made from tobacco products and some plant leaves. Snuff can be used as medication and offers a reduced degree of relaxation and pleasure, but this practice was pushed despite the fact that they were aware of its addictive potential.^20^ Comparing cigarette smoking and ST use in Southern African countries, when using the statistics and health surveys, it appeared that the rate of cigarette smoking is high in the urban areas while ST use (snuff, chewing and pipes) is high in the rural areas. This outcome has been noted in both genders.^21^ The current study’s objectives included defining the socio-demographics of ST users as well as the level of awareness of the health risks associated with the practice of ST; the study sought to ascertain the degree of awareness of the health risks associated with ST use among users in a Pretoria community.

## Methods

### Study design

This was a descriptive cross-sectional study. A piloted, structured and self-administered quantitative survey questionnaire was used.

### Setting

The study was conducted at Ramotse Clinic, a day clinic located in Ramotse (a remote area) which is part of Tshwane region 2. It is 43 km to the north of Central Pretoria, alongside the national road referred to as the N1. Census 2011 showed the estimated population to be around 15 760 in an area of 6003 km^2^.^22^

### Population, recruitment, sampling and estimated sample size

The population of Ramotse, which was approximately 15 760 as per the Census 2011, was used to determine the sample size.^22^ The required sample size for such population was determined to be 376 (*n* = 376) using the net sample size formula also called sample size calculator Raosoft,^23^ with a confidence level of 95% and a 5% margin of error. The final sample size (*n* = 479) was determined by accepting an oversampling during the data collection process because of the participants’ desire to participate. Two retired nurses acted as research assistants (RAs) and were trained by the researcher on how to introduce the study to participants. Every day in the early morning, while patients were waiting for their files at Ramotse Clinic, the first research assistant (FRA) introduced the study (its aim and objectives) to them. This introduction was repeated several times during the day to other new patients who arrived. From the consulting rooms, patients were advised to meet with the second research assistant (SRA), who explained the aim and objectives of the study to each patient for the second time. Those patients (from 18 years and above) who consented to take part in the study were given a consent form to sign, which confirmed their willingness to participate; they then received a questionnaire to complete.

#### Collection tool (questionnaire)

The four authors, based on their prior research experiences, created the questionnaire, which was subsequently tested at the New Eersterus Clinic, one of the clinics in region 2 of Tshwane health district. Nearly 30 km separate it from Ramotse Clinic where the study was conducted. The decision to employ this New Eersterus Clinic was made since it is a day clinic, somewhat remote, similar to the Ramotse Clinic, and their clients share similar sociocultural traits (foods, language, etc.). No data from the pilot was taken into account in the Ramotse study so as not to contaminate the study’s findings. After the pilot study, opinions on the questionnaire were gathered from the participants to make sure that it accurately assessed the health risks related to ST (face validity).^24^ Four authors (three family physicians and one medical officer) also reviewed and adjusted the questionnaire to make sure it was in line with the aim and objectives of the study (content validity).^24^ When the same sample from the pilot study was reviewed and subjected to the same questionnaire a week later, the results were consistent with those from the initial exposure (test-retest reliability).^25^ The questionnaire had two parts: the socio-demographics and the health risks associated with ST use. The questionnaire was written in two languages, English and Setswana, which are the two languages commonly spoken in Ramotse area. The Setswana translation was done by the two RAs who are fluent in both languages. The SRA helped those who could not read and write, but were willing to be part of the study. Data collection took almost seven months (June 2021 – January 2022).

### Data analysis

**FIGURE 1:**
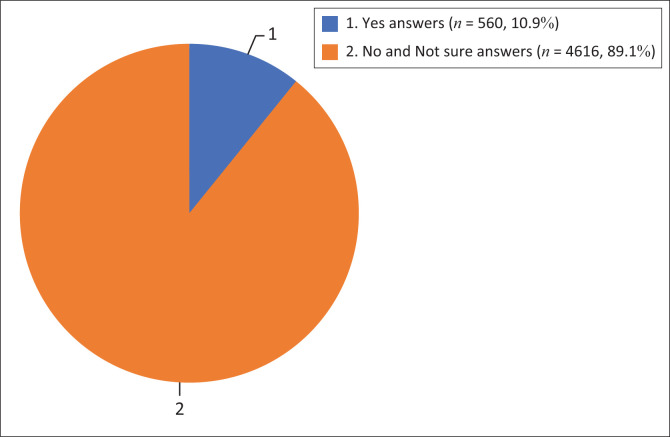
Overall awareness on health risks associated with smokeless tobacco products use.

All data from the spreadsheet was imported to the Statistical Package for the Social Sciences (SPSS) version 28, where descriptive analysis was done. Tables 1 and 2 and Figure 1, that depict frequencies and percentages, are used to present the results. In the second section of the tool relative to the awareness of health risks associated with some health conditions, a ‘Yes’ response indicated that the participant was aware of the relationship between health hazards and the condition, while a ‘No’ or ‘Not sure’ response indicated that the participant was not. The level of awareness is supposed to be indicated by dividing the sum of all ‘Yes’ responses by the total number of responses, whereas the level of unawareness is indicated by dividing the sum of all ‘No’ and ‘Not sure’ responses by the total number of responses.

**TABLE 1:** Socio-demographics.

Variables	Frequency	%
**Age group (years) (*n* = 469)**		
18–29 years	67	14.3
30–39 years	148	31.6
40–49 years	116	24.7
≥ 50 years	138	29.4
Total	469	100.00
**Gender (*n* = 478)**		
Female	371	77.6
Male	107	22.4
Total	478	100.0
**Occupation (*n* = 469)**		
Employed	175	37.3
Unemployed	263	56.1
Learners	3	0.6
Pensioner	28	6.0
Total	469	100.0
**Marital status (*n* = 476)**		
Single	195	41.0
Married	236	49.6
Divorced	23	4.8
Widowed	22	4.6
Total	476	100.0
**Education level (*n* = 475)**		
No formal education	13	2.7
Primary education	107	22.5
Secondary education	270	56.8
Tertiary education	85	17.9
Total	475	100.0
**Chronic illness (*n* = 472)**		
Yes	198	41.9
No	274	58.1
Total	472	100.0
**Method of using smokeless tobacco (*n* = 463)**		
Snuff by nose	338	73.0
Applying between gum and lip	67	14.5
Chewing	58	12.5
Total	463	100.0

**TABLE 2:** Health risks associated with smokeless tobacco use.

Health conditions	Yes		No		Not sure		Total
	*n*	%	*n*	%	*n*	%	
Nicotine addiction	64	13.6	390	83.0	16	3.4	470
Disease of the mouth (leukoplakia)	56	12.4	396	87.6	19	4.2	471
Cancer of the mouth	76	16.2	381	81.1	13	2.8	470
Cancer of oesophagus	57	12.1	388	82.6	25	5.3	470
Cancer of pancreas	36	7.6	398	84.6	37	7.9	471
Early delivery of baby (pregnancy)	41	8.7	398	84.5	32	6.8	471
Increase risks of stillbirth	34	7.2	406	86.2	31	6.6	471
Nicotine poisoning in children and low birthweight	37	7.9	395	83.9	39	8.3	471
Increased risk of death from heart disease	53	11.3	387	82.1	31	6.6	471
Increased risk of death from stroke	49	10.4	385	82.1	35	7.5	469
Increased risk of asthma	57	12.1	386	82.0	28	6.0	471

**Total**	**560**	**-**	**4310**	**-**	**306**	**-**	**5176**

### Ethical considerations

Ethical clearance to conduct this study was obtained from the Sefako Makgatho University Research Ethics Committee (SMUREC) of the Sefako Makgatho Health Sciences University (Ethical clearance number: SMUREC/M/71/2021/: IR).

## Results

A self-administered questionnaire was used and some participants, although being instructed, did not entirely fill out the self-administered questionnaire. As a result, certain missing data were discovered during data analysis. The sample size was 479 (*n* = 479).

Most of the participants were from the age group 30–39 years (148; 31.6%), followed by those aged 50 years and above (138; 29.4%). Most of them were females (371; 77.6%), unemployed (263; 56.1%), married (236; 49.6%), had a secondary level of education (270; 56.8%), did not have any chronic illnesses (274; 58.1%), and used snuff by nose (338; 703.0%), as shown in Table 1.

### Question to the participants

Are you aware that using ST products is a health risk that is associated with the occurrence of health conditions listed below?

The answers ‘No’ and ‘Not sure’ meant that the participant was not aware of the association between health risks and the conditions listed below, while the answer ‘Yes’ indicated that the participant was aware. According to Table 2, the majority of participants were unaware of the use of ST products to be a health risk, therefore, they were also unaware of the associated health conditions as listed in Table 2.

Figure 1 derived from Table 2 shows that 560 (10.9%) of the 5176 responses were ‘Yes’, whereas 4616 (89.1%) were ‘No’.

## Discussion

According to this survey conducted in a Pretoria community, more women than males reported using ST, with snuff being the most common form (Table 1). This finding is similar to that of a Bangladeshi study, with regards to the gender distribution, but differs in terms of the mode of use. In Bangladesh, chewing tobacco was used more than snuff. There is also a similarity when it comes to the area (urban/rural). Although the Tshwane district seems not to have a rural area/community in the entire district, Ramotse where the study was conducted seems to be a remote zone in the district and has a high rate of ST use among women like the rural women in Bangladesh.^8^ This observation, of ST being used more in the rural areas while smoking is used more often in urban areas, was also highlighted in a Southern African survey.^18^ The Pretoria study deviates from the WHO viewpoints on gender in that it depicts men as using ST at higher rates and refers to smoking as a sign of masculinity.^2^ Despite the fact that primarily women use ST products, according to a recent Pretoria study, South Africa as a whole has a more complex picture when it comes to this topic. Some papers showed greater interest among primary school students,^4^ whereas other studies focused more on women’s childbearing.^5^

In Ramotse, Pretoria, the majority of participants were from the age group 30–39 years (Table 1). This is inconsistent with what was observed in Wonju, Korea, where the majority of the ST users recruited were aged under 30 years.^7^ This discrepancy might be explained by participant availability, particularly, as it was in the Pretoria trial. One of the research projects cited in this paper also shows that teenagers in South Africa are engaged in ST use^4^; and this is one of the study’s limitations because children under the age of 18 years were not taken into account.

Although married women in Madaripur, Bangladesh, are also involved in ST use, they differ from the group in Pretoria in that in the latter there is no association with any of their sociodemographic data, while for the women in Bangladesh, there are associations.^8^ This inconsistency is also observed in relation to a study conducted in Ethiopia, where the use of ST was strongly associated with the lifestyle of the community. Here, religion was considered as one of the defensive factors, and a high degree of social pressure was dependently connected to the practice.^17^

The use of tobacco products has been linked, in the literature, to a greater number of diseases and a higher death rate^2^ in contrast to the evidence in the Pretoria trial. In the literature, it has been described that more health conditions and a high death rate have been established as related to the use of tobacco products.^2^ Globally, the low- and low-middle income nations pay the most price for this use of ST, especially in the rural communities. This is in line with what was stated about Bangladesh, where it is anticipated that this practice will lead to significant noncommunicable diseases.^8^ Sadly, this runs counter to the Pretoria study, where only 58% of respondents claimed to have chronic illnesses (Table 1), and since the questionnaire did not specifically ask about medical problems, it was challenging to connect the health risk with the disorders.

Smokeless tobacco use increases the risk of heart and brain conditions, that most of the time lead to death, as described in the United States of America and Sweden.^4^ The practice of ST use was linked to some of the chronic and terminal health conditions such as oral and pharyngeal cancers, strokes, periodontal illnesses, erectile dysfunction, stillbirth and low birthweight of new-borns, as stated in a study carried out in Sri Lanka.^6^ In Sweden, many cigarette smokers opted for ST use, with the idea that snus (snuff) or ST causes less harm than the combustion of tobacco products. Surprisingly, a study done in the country among users of snus or snuff and also users of cigarette smoking found that participants in both groups had an increased risk of diabetes mellitus type 2.^10^ Same observation of high risk of diabetes mellitus was also noted in Australia among pregnant women who chewed ‘pituri’^11^ and in Venezuela among users of ‘chimo’.^12^ In Asia, asthma, diabetes mellitus, dyslipidaemia, abortion and low birthweight were described in those who used ‘paan’ and ‘gutka’ (ST products).^10^ In India, ST use was associated with conditions such as oropharyngeal-oesophagus cancers and ischaemic heart disease. This study was able to determine the level of awareness of the aforementioned association in Pretoria and discovered that participants’ ignorance of the relationship between health risks and health issues is common in both Pretoria and Nigeria.^17^

### Strengths and limitations

Although these are primary data, the findings cannot be generalised because not all Ramotse ST users and no individuals under the age of 18 years participated in this study. Lack of measurement for heated tobacco products is also a limitation for this current study, hence it is advised that future studies evaluate heated tobacco product use.

## Conclusion

This study has demonstrated a poor awareness of health risks associated with ST use among users in a Pretoria community; as a result, health education at various levels of the community (clinic, schools, ward-based outreach team or WBOT, etc.) could be one strategy for resolving the problem.
